# An evaluation model for interactive gaming furniture design based on parent-child behavior

**DOI:** 10.1371/journal.pone.0302713

**Published:** 2024-06-07

**Authors:** Yanfeng Miao, Xuefei Gao, Weiye Jiang, Wei Xu

**Affiliations:** 1 College of Furnishings and Industrial Design, Nanjing Forestry University, Nanjing, China; 2 Jiangsu Co-Innovation Center of Efficient Processing and Utilization of Forest Resources, Nanjing, China; Prince Sattam bin Abdulaziz University, SAUDI ARABIA

## Abstract

This study takes the parent-child game behavior of children aged 3~6 and their parents as the research object, and extracts and summarizes the user behavioral needs of parents and children when they use game-based furniture together by using the questionnaire research method, observation method, and interview method. Based on the KJ method, 16 behavioral demand indicators were compiled by five furniture design students to construct a user behavioral demand system. In addition, AHP and entropy weight method were used to solve the user behavioral demand weights from subjective and objective perspectives in this study. Twenty experts and designers in this research field scored the indicators two by two and solved the subjective weights of user behavioral requirements according to the AHP algorithm. A seven-level Likert scale was used to design the questionnaire and distribute it to the parents of children aged 3–6 to fill in, and the 121 valid questionnaires obtained were used as raw data for entropy weighting to obtain the objective weights of user behavioral needs representing the opinions of interactive game-based furniture users. Finally, with 0.4 as the proportion coefficient of subjective weights, the subjective and objective weights were weighted to get the comprehensive weight value of each demand. The results show that the eight items with higher weights for user behavioral needs include: firm and stable, safe in use, comfortable for both parents and children, holding behavior by human-machine dimensions, able to sit on the ground and play, able to play face-to-face, easy to find for picking up, and sufficient operating space. In general, parent-child interactive game furniture firstly needs to meet the user’s needs for safety and comfort, and secondly needs to meet the user’s needs for the state of the game posture and the furniture size to meet the needs of the fetching and storage posture and the game space. The fuzzy comprehensive evaluation model established based on these needs can take into account the opinions of design experts and users at the same time and put the needs of children and parents in an equally important position so that the design of children’s play furniture can tend to meet the needs of parents and children when they use it together, and to promote parent-child interaction and the healthy growth of children.

## Introduction

For children in the preschool stage (3–6 years old), playing games is their primary way of life. Games help them develop motor skills, cognitive abilities, and social and emotional abilities, some of which are acquired through repeated operations in play. Meanwhile, preschool children aged 3–6 are in a critical period from emerging to developing self-awareness. A family is an important place for the early socialization of children [1], and positive parenting behavior is central to efficacious interventions for child conduct problems [2]. Parent-child relationships significantly impact children’s physical and mental health, social cognition, emotional cognition, and social behavior development [3]. Therefore, conscious guidance from parents during play can particularly impact children’s personality, cognition, and actions. Parent-child interactive play is mainly done through furniture, and suitable furniture design can also promote parent-child interaction. For example, comfortable chairs that can restrain children’s active nature can ensure that parent-child reading is carried out smoothly and effectively. Stacking furniture that requires parents to help children move can satisfy parents’ need to enjoy the warmth between parents and children. Appropriate scale tables or stools can reduce the physical fatigue of parents accompanying children to do desktop games, enhance the parents’ interest in participating in the game, and prolong the parent-child interaction time.

In the current study, the research on children’s game furniture mainly focuses on safety, growability, and fun, mostly from the children’s psychology, physiology, and behavior, combined with the children’s game theory to put forward the corresponding design principles, and for the modeling, function, structure, color, material and the combination of the way of the game and the combination of the design elements of children’s furniture analysis. Children’s furniture should correspond to children’s game instincts, and specific game activities should be included in children’s furniture design [4]. Most of the research on parent-child interaction has been conducted on the psychological discipline and parent-child relationship, and relatively few studies have been undertaken on design. Scholars in various countries mainly analyze the influence of the parent-child relationship on children and parents from the psychological level. One is to explore the overall dimension of the parent-child relationship through psychological questionnaires, statistical analysis, and theoretical deduction. The other is to examine the influence mechanism behind the behavioral characteristics of the parent-child relationship through more rational methods such as observation and recording of parent-child behavior. Although there are very few studies related to parent-child interactive game-based furniture, the actual exploration of parent-child interactive products has been more mature in the field of toys, which can be mainly divided into several categories of competitive, guiding, cooperative, and accompanying, and the related toy products have a more apparent age classification. In the theoretical study of parent-child interaction furniture design, scholars have proposed that the interaction experience of preschool children comes from two aspects: the comfort of furniture use and the pleasure of interaction. Children’s performance also determines parents’ interactive experience, and in the process of interaction, most parents tend to ignore their own experience for the sake of children’s interactive experience and the developmental results brought by interaction. This phenomenon is mapped to the actual products on the market, manifested in the current children’s use of game furniture is biased towards children’s games, and parents are often accompanied by an uncomfortable position to play, which significantly affects the effect of parent-child interactive games. If the game-type furniture is more concerned about parent-child interaction properties can adequately improve the comfort of both parties, it is easier to create a relaxed and happy family game atmosphere, thus giving full play to the role of parental guidance and promoting the children to have a more healthy and beautiful personality, and therefore the study of parent-child interaction game-type furniture is of great significance.

This study focuses on a three-member family of parents and children living in the city. The parents emphasize children’s education and play training, and the housing conditions are medium to large-sized. They are more concerned about the children’s physical and mental health and development after they start kindergarten, so they often purchase some household items related to children. The parent-child interactive play furniture referred to in the study refers to the furniture used by parents and children during play interaction, usually in the form of play media or props, to meet the needs of parents and children in an indoor environment. The study explores the behavior of children aged 3–6 and their parents during play interaction, exploring user needs from the perspectives of parent-child preferences and parent-child interaction behavior. The user behavior needs during parent-child play interaction are proposed to compensate for the current research’s shortcomings. The study also considers the importance of individual user behavioral demand factors based on an AHP-entropy weighting theoretical approach.

### Outline of the design model

**Step 1.** Classifying preschool children’s games to provide clear game classification standards for user behavior needs research.
**Step 2.** Analyze the product information related to children’s play furniture in the market and classify it in a standardized manner to establish the relationship between games and furniture products. It is convenient for investigating users to understand the positioning of parent-child interactive game furniture.
**Step 3.** Obtain users’ furniture preferences and behavioral needs through questionnaires, household observations, and interviews.
**Step 4.** Using the Analytic Hierarchy Process (AHP), the subjective weight values of user behavior needs are obtained through industry expert scoring.
**Step 5.** Obtain data in the form of a questionnaire from the user’s evaluation of the importance of indicators, and use the entropy weight method to calculate the objective weight value of user behavior needs. Step 6. Calculate the comprehensive weight values of user behavior requirements and sort them.

## Literature review

Literature related to parent-child interaction mainly focuses on the three classifications of social sciences, science and technology, and life sciences and biomedical sciences, and research on parent-child play focuses on cross-cultural research on parent-child play, psychotherapy, parental roles, and factors influencing parent-child play. At the same time, there is a relative lack of research under the classification of humanities and arts. Factors influencing parent-child interactions mainly include family background, parenting style, and parent-child gender. Family background covers a range of factors, such as parental income and occupation. Some studies have shown that fathers in families with above-medium income levels are more likely to believe that play is suitable for young children and are more likely to engage in parent-child play with them; fathers who work full-time are less likely to recognize and participate in play than fathers who work part-time [5]. There is a tendency for the time gap in child growth and development activities between parents with high and low levels of education to widen [6]. For parenting styles, some scholars have classified the mode of parental treatment of children. After observing and analyzing more than one hundred families in the U.S., some scholars classified parenting styles as authoritative, authoritarian, spoiled, and neglectful and concluded that among them, authoritative parents have authority in their children’s minds but respect and understand their children. It is the kind of parenting style that is the most favorable to their children [7]. From the perspective of parental gender, differences in parental gender can lead to different influences on children. The quality of the mother-child relationship predicts self-affirmation in children, while the father-child relationship is more predictive of children’s self-esteem, behavioral performance, and social competence [8]. From a child’s gender perspective, differences in children’s gender lead to differences in parental treatment. Parents of girls are more likely to engage in social play with girls, while parents of boys are more likely to engage in active play with them [9]. There is a great deal of variation in mothers’ language styles during parent-child play, with affectionate declarative sentences (e.g., supportive language) dominating communication with their daughters and an authoritarian tone (e.g., commands) being less likely to be used when communicating with their sons [10]. Some scholars also believe that parents will use more indirect language strategies (such as more participation and completing games according to children’s requirements) to teach their daughters during parent-child games [11]. These general patterns have emerged from researchers’ investigation and analysis of different family situations, such as the correlation between the level of family income and the father’s approval of play, and the impact of parenting styles on children’s behavior. However, although some critical findings have been made, there are still some challenges and shortcomings. For example, while there are different ways of categorizing parenting styles in research, how to accurately assess the effects of parenting styles and their long-term impact on children’s development needs to be further explored. In addition, studies have found some differences in the relationship between parents and children’s gender, but explaining these differences and their specific impact on family interactions still needs more in-depth research. What is certain, however, is that families with above-middle incomes, high levels of education, and families in which parents are actively involved in the development of their children place greater emphasis on parent-child play activities and have a higher demand for such activities.

Several product-related design elements are usually involved in the process of product design [12]. Considering all the design elements of a product and selecting the best design solution has a crucial impact on the product’s market share and user satisfaction. Multi-criteria decision-making models give a corresponding solution to this problem, i.e., using the available decision information to rank or select the best of the given finite number of decision alternatives. In the decision-making process, the weights of the attributes are generally determined first. Multi-baseline decision-making methods can be classified into two categories based on the stage of their main application. One category is for methods used to solve for factor weights, such as AHP, entropy weight method, dominance assignment method, etc. The other category is the methods used to prioritize solutions, such as TOPSIS, Grey Correlation Method, VIKOR Method, ELECTRE Method, etc. In practical research, depending on the needs of a particular problem, two or more evaluation methods can be used alone or in combination for analysis. Multi-criteria decision-making methods for solving factor weights can provide effective indicators for project or program evaluation, and the traditional systematic evaluation methods are mainly carried out through subjective analytical assignments or objective data assignments. The most widely used subjective analytical empowerment belongs to the AHP. Collecting data in the form of interviews and questionnaires, analyzing the collected data through the factor analysis method, which can determine the potential components or dimensions between the indicators, identifying the main factors, and determining the weights of the factors or dimensions by the Fuzzy Hierarchical Hierarchical Analysis Method (FAHP), and combining with the statistical method of Structural Equation Modelling (SEM), which can verify the consistency between the theoretical model and the observed data, and determine the effective indicators in the actual project [13]. The entropy weight method (EWM) is an objective assignment method for determining the weights of each criterion in multi-criteria decision-making, which can deal well with the correlation and information contribution between each criterion based on actual data [14]. The entropy weight method is used in conjunction with the Soil and Water Assessment Tool (SWAT) to evaluate, and screen management strategies (BMPs) to reduce environmental pollution and resource wastage by weighing efficiency and cost-effectiveness and the hybrid use of the SWAT model and entropy weighting method can economically help to improve existing and future BMPs [15]. The packet network analysis (DEA) and entropy weights methods share similarities in that both rely on input data and can be used to support the decision-making process. However, DEA is a linear programming method mainly used to determine the relative efficiency of multiple decision units rather than the criteria weights [16]. Hierarchical Analysis of Hierarchy (AHP) and ELECTRE methods are subjective decision-making methods, and their combination can improve the accuracy and reliability of decision-making. AHP determines the relative importance of each criterion by constructing a hierarchical structure and comparing the two. ELECTRE determines the priority among the alternatives and selects the best alternative based on the weights of the criteria determined by AHP and the actual performance of the alternatives [17]. Similarly, TOPSIS is used to select the optimal solution, which determines the best solution based on a comparison of selection similarity with the best solution (positive ideal solution) and the worst solution (negative ideal solution) [18]. TOPSIS can be used with Failure Mode and Effects Analysis (FMEA) in accident prevention and protection mechanisms. By using FMEA as a risk identification tool and introducing fuzzy ideas considering the fuzzy nature of risk evaluation indicators, the fuzzy risk priority number (FRPN) can be calculated, followed by TOPSIS to identify and prioritize error and risk factors, which can effectively assess the risk level [19]. VIKOR is commonly used to solve decision-making problems with multiple attributes and candidate solutions [20]. Some researchers have proposed a new VIKOR method based on the Q single-valued neutrosophic set, mainly used to discuss the optimal choice model in cyber warfare and emphasize the scientific and rational nature of the method when conflicting attributes are involved [21]. Although many methods are used to optimize decisions and support the decision-making process, the ultimate goal of the selection and combined methods is to solve problems, so different methods are used for different problems. In this study, we focus on the user needs of parent-child interactive game-based furniture. In other words, we explore the evaluation criteria of parent-child interactive game-based furniture based on user satisfaction, so only the first stage of the multi-criteria decision-making process, i.e., solving for the factor weights, is involved. In design-related fields, there is a greater tendency to determine attribute weights through subjective assessments by experts or decision-makers. This method is highly subjective and prone to deviations between decision-making solutions and user expectations. The objective assignment method does not have any subjective factors and relies on assigning the raw data of the indicators to calculate the weights, which is highly objective and accurate. Still, when used alone, the evaluation results may not align with the expectations due to the small number of indicators or inaccurate calculation of the indicator data.

## Theoretical background

To effectively design and evaluate parent-child interactive game-based furniture, we will use a comprehensive approach to determine the weights of user requirements and their ranking. Realizing that relying solely on expert scoring or autonomous user ratings may have subjectivity or objectivity bias, we propose an evaluation model that combines subjective and objective assignment methods. This integrated approach aims to ensure that the evaluation results adequately reflect users’ actual needs while having a high degree of feasibility and practicality, thus providing more practical guidelines for the design and evaluation process. A new evaluation model consisting of the hierarchical analysis method (AHP) and the entropy weight method (EWM) is used in this study.

The Analytic Hierarchy Process (AHP) provides a mathematically structured method for comparing two items’ relative attributes and importance [22]. This structured approach helps decision-makers better understand the problem’s complexity and more accurately assess the relationship between different factors [23]. In our study, we used a unified evaluation system for subjective and objective assignments, and this structured evaluation system helps users to understand our problem better and thus make their scoring more accurate. In addition, AHP uses a two-by-two comparison to determine the relative importance between different factors, and the decision maker only needs to compare the significance between two factors without directly giving a specific numerical score, which can reduce the burden of the evaluation process [24]. Compared with other objective weighting methods, such as Data Envelopment Analysis (DEA), Principal Component Analysis (PCA), and Gray Correlation Analysis (GRA), entropy weighting is relatively simple and intuitive and is not affected by the distribution of the data which can better reflect the differences between the indicators, etc. The combination of AHP-EWM weighting is often used in environmental assessment, risk decision-making, and project selection and evaluation and has been effectively applied [25]. In product design and marketing, it can determine the importance of product characteristics, assess market demand, and guide product design and marketing strategies. Therefore, the AHP-EWM combined evaluation model is effective for this study.

The process of AHP-EWM combination assignment in this study is as follows: children’s furniture designers or experts score the indicators two by two according to the requirements of AHP, and then the target users of parent-child interactive game-type furniture evaluate the indicators through questionnaires, and then the obtained data are subjected to the AHP and entropy weighting algorithm operations and weighting treatment respectively, and finally, the weights of each indicator are ranked.

### The analytical hierarchy process (AHP)

AHP can divide the hierarchy of a problem into multiple different influencing factors, and the steps to calculate the weight of demand factors using AHP are as follows:

**Step 1.** Build a judgment matrix. Expert decision-makers use the 1–9 evaluation scale method as a Delphi survey evaluation method to assign the importance of two indicators to a specific numerical value and construct a judgment matrix, as shown in Eq (1).


$$A=(a_{ij})=[\begin{array}{cccc} a_{11} & a_{12} & \cdots & a_{1n}\\ a_{21} & a_{22} & \cdots & a_{2n}\\ \cdots & \cdots & \cdots & \cdots \\ a_{n1} & a_{n2} & \cdots & a_{mn} \end{array}]$$ (1)

In the formula, *c*_*ij*_ is the vital value for the criterion layer obtained by comparing factor *i* and factor *j*, and *c*_*ij*_>0, *c*_*ij*_ = 1/*c*_*ji*_.

**Step 2.** Normalize each column of matrix C by Eq (2).


$$a_{ij}=\frac{c_{ij}}{\sum_{i=1}^{n}c_{ij}}$$ (2)

**Step 3.** The normalized matrix is summed by rows using an approximate method of finding the eigenvectors of the judgment matrix by Eq (3).


$$\bar{w_{i}}=\sum_{i=1}^{n}a_{ij}$$ (3)

**Step 4.** The subjective weights of each behavioral demand factor can be obtained by finding the weight vector by $w^{1}=(w_{1}^{1},w_{2}^{1},\cdots ,{w_{n}^{1})}^{T}$ Eq (4).


$$w_{i}=\frac{\bar{w_{i}}}{\sum_{i=1}^{n}\bar{w_{i}}}$$ (4)

**Step 5.** Calculate the maximum characteristic root *λ*_max_ to break the consistency of the matrix. The maximum characteristic root $\lambda_{max}=\frac{1}{n}\sum_{i=1}^{n}\frac{{\left(Cw\right)}_{i}}{w_{i}^{1}}$, (*Cw*)_*i*_ denotes the *i*-th element of vector *Cw*. The final consistency test is based on consistency indicator $CI=\frac{\lambda_{\max}-n}{n-1}$, random consistency indicator *RI*, and consistency ratio *CR*. When *CR* = 0, there is complete consistency; When *CR* = *CI*/*RI*<0.1, it is considered that *C* is a satisfactory consistency matrix, and the obtained weights have validity. Conversely, it will be necessary to modify or discard this judgment matrix to some extent. *RI* is obtained by looking up the table based on the order of the matrix.

### Entropy weight method

The entropy-weight method is based on Shannon entropy, initially developed by Shannon [26]. According to the relevant entropy characteristics, the entropy value can be used to determine the degree of dispersion of a specific indicator. The greater the degree of dispersion, the greater the weight value, and the more significant the impact on a comprehensive evaluation. The steps to calculate the weight of demand factors using the entropy weight method are as follows:

**Step 1.** Build the original matrix *B*. Calculate the weight of *m* users and *n* evaluation indicators to obtain a data matrix by Eq (5).


$$B={\left(b_{ij}\right)}_{m\times n}=[\begin{array}{cccc} b_{11} & b_{12} & \cdots & b_{1n}\\ b_{21} & b_{22} & \cdots & b_{2n}\\ \cdots & \cdots & \cdots & \cdots \\ b_{m1} & b_{m2} & \cdots & b_{mn} \end{array}]$$ (5)

**Step 2.** Standardize the data. Process the raw data *b*_*ij*_ to eliminate differences between indicators. Then we get *P* = (*p*_*ij*_)_*m*×*n*_, where *p*_*ij*_ is the standard value of the *i*-th indicator and the *j*-th evaluation object.

Calculate *p*_*ij*_ by formula Eq (6) for smaller and better indicators.


$$p_{ij}=\frac{\left({\left\{x_{ij}\right\}}_{\max}-x_{ij}\right)}{\left({\left\{x_{ij}\right\}}_{\max}-{\left\{x_{ij}\right\}}_{\min}\right)}$$ (6)

Calculate *p*_*ij*_ by formula Eq (7) for larger and better indicators.


$$p_{ij}=\frac{\left(x_{ij}-{\left\{x_{ij}\right\}}_{\min}\right)}{\left({\left\{x_{ij}\right\}}_{\max}-{\left\{x_{ij}\right\}}_{\min}\right)}$$ (7)

In the formul, {*x*_*ij*_}_max_ represents the maximum value of *x*_*ij*_ in column *j*; {*x*_*ij*_}_min_ represents the minimum value of *x*_*ij*_ in column *j*.

**Step 3.** Normalize data by Eq (8).


$$h_{ij}=\frac{p_{ij}}{\sum_{i=1}^{n}p_{ij}}$$ (8)

**Step 4.** Calculate the entropy value (*E*_*i*_) of the second indicator $E_{i}=\frac{-\sum_{j=1}^{m}h_{ij}\ln h_{ij}}{\ln m}$, *E*_*i*_∈[0,1], and if *P*_*ij*_ = 0, define $\underset{P_{\mathrm{ij}}\to 0}{\lim}P_{ij}\;\ln\;P_{ij}=0$.

**Step 5.** Calculate the entropy weight. $B_{i}=\frac{\left(1-E_{i}\right)}{\sum_{i=1}^{n}\left(1-E_{i}\right)}$, and *B*_*i*_∈[0,1], $\sum_{i=1}^{n}B_{i}=1$.

Finally, the combined weights are obtained by combining subjective and objective weights. The combined weight coefficient value can be obtained by adjusting the subjective weight coefficient value *w*_*i*_ with the entropy weight value *α*_*i*_ through formula Eq (9).


$$W=\alpha w_{i}+(1-\alpha )B_{i}$$ (9)

## Case study

### Pre-school children’s game classification

Since children’s physical and mental states develop rapidly during the 3-6-year-old stage, there is some variability in the level of children’s play in the three age groups in between. This study used the theoretical references of Piaget’s theory of play, the Guidelines for Learning and Development of 3-6-year-olds, the Montessori pedagogy, theories related to children’s play, and the classification of play behaviors in preschool education. Kindergarten is the primary place for children aged 3–6 to engage in play activities, and the play activities that take place in kindergarten can represent all the play activities that children at this stage are involved in, which means that parent-child interactive play is also included. We observed and recorded the play behaviors of Chinese children in kindergartens in the preschool age group and had in-depth conversations with kindergarten teachers on topics related to children’s play activities. After discussion and deliberation among the research team, all children’s games were categorized, and the standard features of each category were extracted as the name of the category. The purpose of classifying children’s games is to provide clear criteria for categorizing games in the children’s game furniture and questionnaire. In the questionnaire, the three age groups were given the appropriate game categories to prevent parents from choosing games beyond the children’s age range, which may have an unscientific influence on the design of the furniture.

Table 1 shows the types of games currently suitable for children’s current age group needs and development level, which can be classified into seven types at the age of 3–4, six types at the ages of 4–5, and 5–6. All types of games can be divided into seven categories: sports games, painting and handicraft games, sensory cognition games, music/performance games, character games, puzzle games, and structural games. Due to the rapid changes and development of children’s physiology and psychology, single sensory cognition games have faded out of children’s game choices after age 4, so sensory cognition games are excluded from game classification after age 4. In this study, we conducted preliminary research on children’s game furniture on the market. After understanding and analyzing the use and function of each product, we found that the general forms of games that can be combined with furniture are sports games, drawing and handcrafting games, sensory, cognitive games, role games, intellectual games, and structural games, and the music performance games don’t need to use furniture.

Sports games: Games with the theme of developing basic movements such as running, jumping, throwing, climbing, etc.

Painting and handicraft games: A painting or manual game played using brushes, paper, cutting tools, and pasting tools.

Sensory cognition games: Cognitive games aimed at exercising visual, olfactory, tactile, auditory, and taste senses.

Music/Performance games: Performance games such as singing, playing, recitation, dancing, etc. with or without props;

Character games: A game that creatively reflects the surrounding life through role-playing, relying on imitation and imagination;

Intelligence games: Calculating the relationship between the number of objects or playing games according to rules;

Structural games: A game that uses structural materials such as building blocks, plastic blocks, sand, and mud to build.

**Table 1:** Criteria layer matrix, weights, and consistency test results.

Game Categories	3–4 years old	4–5 years old	5–6 years old
**Sports games**	1: Such as ball tossing, jumping (sports) class games) 2: Eagle catching chickens, jumping lattice (transport Motivational rules game)	1: Drill holes, throwing and catching balls, chasing/ dodging Run	1: Climbing the net/frame, dodging the ball/sandbag, hitting the ball
**Painting and handicraft games**	3: Such as doodling, paper cutting, collage	2: Painting, cutting simple shapes, paper folding, kneading dough	2: Painting, cutting and pasting art works, kneading dough
**Sensory cognition games**	4: Material tactile cognition games 5: Object shape cognition games (e.g. shape matching)		
**Music/Performance games**	6: Singing, dancing, beating the beat Children’s songs/nursery rhymes/stories	3: Singing, dancing, beating the beat Children’s songs/nursery rhymes/stories	3: Singing, dancing, beating the beat Children’s songs/nursery rhymes/stories
**Character games**	7: Simple character-playing games (Play Barbie, Transformers)	4: Playing games that mimic real life (themes such as family, hospital, etc.)	4: Dressing up/character-playing games
**Intelligence games**	8: Compare the number of objects using a one-to-one correspondence way	5: Rule games (chess, cards, etc.) 6: Category, sort, and combination games (categorize objects by size, sort, puzzle, etc.) 7: Comparing the number of objects by counting	5: More complex rule-based games (chess, competitions, etc.) 6: Logic games such as classification/sorting/reasoning (puzzles, etc.) 7: Adding and subtracting through physical objects
**Structured games**	9: Stacking geometry simply (Stacking Lego simply etc.)	8: Stacking geometry (blocks, Lego, etc.)	8: Disassembling or making toys, creative assembly (using building blocks, LEGO)

### Classification and characteristics of children’s play furniture

In order to enable survey users to have a certain understanding of the current situation of children’s game furniture at a macro level, facilitate the parent group to better understand the functions and characteristics of children’s play furniture under various types, and provide reference standards for survey users to express their needs accurately, it is necessary to conduct market research before conducting user demand research, classify children’s game furniture categories based on market research, and summarize its characteristics.

Due to the difficulty in researching children’s game furniture products in offline shopping malls and stores, 48 pictures of children’s game furniture were collected through online research ways (including brand official websites, Taobao, and global furniture sales websites), including 38 brands worldwide. There is no standardized classification method for children’s game furniture. Therefore, in this study, based on the essential usage form of the product, it is classified into stacking and combining category (A), table and chair category (B), riding category (C), scene simulation category (D), and space construction category (E). Statistical analysis was conducted on 48 children’s play furniture products, including brand/design, materials, structure, load-bearing game forms, and other functions. Table 2 shows the game types, material preferences, structural preferences, and other functions beyond the game functions that each of the five types of children’s game furniture analyzed in the study can carry. The data in the table refers to the percentage of furniture occupying the attribute option under a specific type of furniture in the total quantity of that type.

**Table 2:** Characteristics of the attributes of various children’s play furniture.

Classification	Games that can be hosted	Structural preferences	Material preference	Other functions
**Stacking and combining category**	structural games (100%), painting and handicraft games (40%), character games (40%), sports games (20%)	combined structure (100%)	fabric (60%), wood (40%) and others (20%)	
**Table and chair category**	structural games (85%), intellectual games (75%), painting and handicraft games (70%)	monolithic structure (45%), assembled (25%), combined (25%), folded (5%)	wood (80%), plastic (35%), fabric (25%)	storage function (60%), size adjustable function (25%)
**Riding category**	character games (75%), sports games (62.5%)	monolithic structure (75%), assembled (12.5%) and combined (12.5%)	plastic (50%), wood (25%), fabric (25%), other (25%) and metal (12.5%)	size adjustable function (12.5%), storage function (12.5%)
**Scene simulation category**	character games (90%), sports games (10%)	combined structure (60%), folded (20%) and monolithic (20%).	wood (80%), fabric(30%)	storage function (10%)
**Space construction** **category**	sports games (80%), character games (20%)	assembled (60%), monolithic (20%) and combined (20%)	wood (80%), fabric (80%), metal (20%) and other materials (20%)	

Only two children’s play furniture explicitly surveyed pointed out that it can meet the needs of parent-child interaction games. In contrast, other furniture is mostly designed only to meet the needs of children. Parents also use children’s furniture during companionship, and the size is often not suitable for parents. The analysis of the functions of children’s play furniture mainly focuses on the types of games and additional functions the furniture can meet. Regarding the game functions, music performance games that are unsuitable for combining with furniture and sensory, cognitive game forms that are only suitable for 3-year-old children are excluded. Statistical analysis was conducted on whether sports games, painting and manual games, character games, intelligence games, and structural games can be met. It can be found that the types of games that the product can meet have a strong correlation with coding (basic usage form).

The game type targeted by the stacking and combining (A) is focused on structural games, which utilize the combination of furniture blocks for structural transformation. When the product shifts to the form of tables and chairs during the form transformation, the suitable game form of the product tends to be similar to the functions of tables and chairs.

Table and chair furniture (B) focuses on drawing, handcraft, intelligence, and structural games. Currently, table and chair furniture is primarily a platform for storing game props and toys and is rarely combined with game forms.

Riding (C) is a type of game that focuses on character games and sports games due to its easy-to-create story scenes, role-playing atmosphere, and the use of wheels and swing forms.

The game form targeted by scene simulation(D) also focuses on character games, including children simulating the lives of others, children simulating adults’ life and work, and presenting play scenes. The first two types of furniture are combined with game props that match the size of children’s bodies to construct life scenes, while the third type mainly uses patterns to create some motion rules, road rules, etc.

The game form targeted by space construction (E) focuses on sports games, mainly providing children with a spatial atmosphere of drilling, crawling, and hiding, satisfying their exploration desire and ’hiding ’psychology.

The main additional furniture functions under all categories are size adjustable and storage, with furniture with size adjustable size function accounting for 12.5% and furniture with storage properties accounting for approximately 29.2%.

### Obtain user behavior requirements

User behavior analysis is an in-depth exploration and summary of factors, such as the reaction forms and usage habits of users during the process of using a product, in order to improve and guide the product based on the problems and needs of users during the use of the product or service experience. Studying user behavior in the design of parent-child interactive gaming furniture products can enable parents, children, and parents to achieve expected and better user and interaction experiences. User demand analysis mainly aims to extract commonalities among most families while exploring special individual needs. Children’s game needs, parents’ usage needs, and parent-child interaction needs are all modules that need to be researched and analyzed for parent-child interaction game furniture.

## Methods

The research on the human, behavioral, and environmental elements of parent-child play was conducted mainly in the form of a large-sample quantitative study-questionnaires, and a small-sample qualitative study-field household observations and interviews. The questionnaire survey mainly focuses on the questions that target users can easily answer and provide feedback on in their daily lives and games, mainly related to preferences and habits. Observation and interview research aim to explore users’ needs that they do not quickly notice during the game behavior process. The questionnaire was rolled out before and after the interviews for data collection, which began on December 1, 2022 and ended on January 5, 2023. Respondents were required to read the written research informed consent form before completing the questionnaire, and only those who agreed were allowed to complete the questionnaire. The behaviors of children aged 3–6 years old during play interactions with their parents needed to be observed in this study, and the minors’ participation in this study had sought and obtained consent from the children’s parents and verbal informed consent for the study was obtained prior to the observation, as evidenced by the fact that the respondents agreed to record parent-child interaction behaviors with a camera.

The target audience for questionnaire distribution is children aged 3–6 and their parents. The questionnaire was designed first to understand the types of games that 3-6-year-old children participate in with their parents to provide a basis for selecting the play scenarios to be observed during the field observation phase of the research. The big difference in height and size between children and adults will inconvenience the communication between parents and children, and in parent-child games, parents often adopt a posture that accommodates children’s height and facilitates interaction as much as possible. Different postures will require different furniture and human-machine dimensions, so in the questionnaire, parents and children were asked about their favorite playing postures. To ensure the validity and reliability of the study’s results, the parents’ questions were presented in plain text, and corresponding pictures accompanied the questions involving children as explanations. The questionnaire was collected using a combination of online distribution and face-to-face completion. Fifty questionnaires were distributed online, mainly targeting families of 3-6-year-old children who could not fill out the questionnaire in person, and detailed filling rules were explained before filling out. The remaining 150 questionnaires were filled out face-to-face by professionals who had undergone unified interpretation of the questionnaire at the entrance of kindergartens or households in cities such as Nanjing, Suzhou, and Zhenjiang.200 questionnaires were distributed, and a total of 156 valid questionnaires were collected, with an effective rate of 78%. The required sample size was determined as the minimum effective feedback response. Frequency analysis, multiple response frequency analysis, chi-square goodness of fit tests, and cross-tabulation chi-square tests were carried out on the data using SPSS software.

To ensure the validity and reliability of the study, objective parent-child play behaviors occurring in an indoor setting throughout the day in these families were observed and recorded with a camera using a non-participant observation format that would not impact parent-child daily interaction behaviors. Interviews are used to supplement the deficiencies in the questionnaire and observations, making it easier to export product pain points. We selected all parents of children from 10 households who participated in the observation survey, and some participated in the questionnaire survey as the interview subjects. In the interview, face-to-face, phone, and WeChat methods were used. Under the premise of assuming a parent-child interactive game environment, a series of questions were asked about game furniture, game environment, children’s behavior, and parents’ behavior.

### User pain point

**Fig 1:**
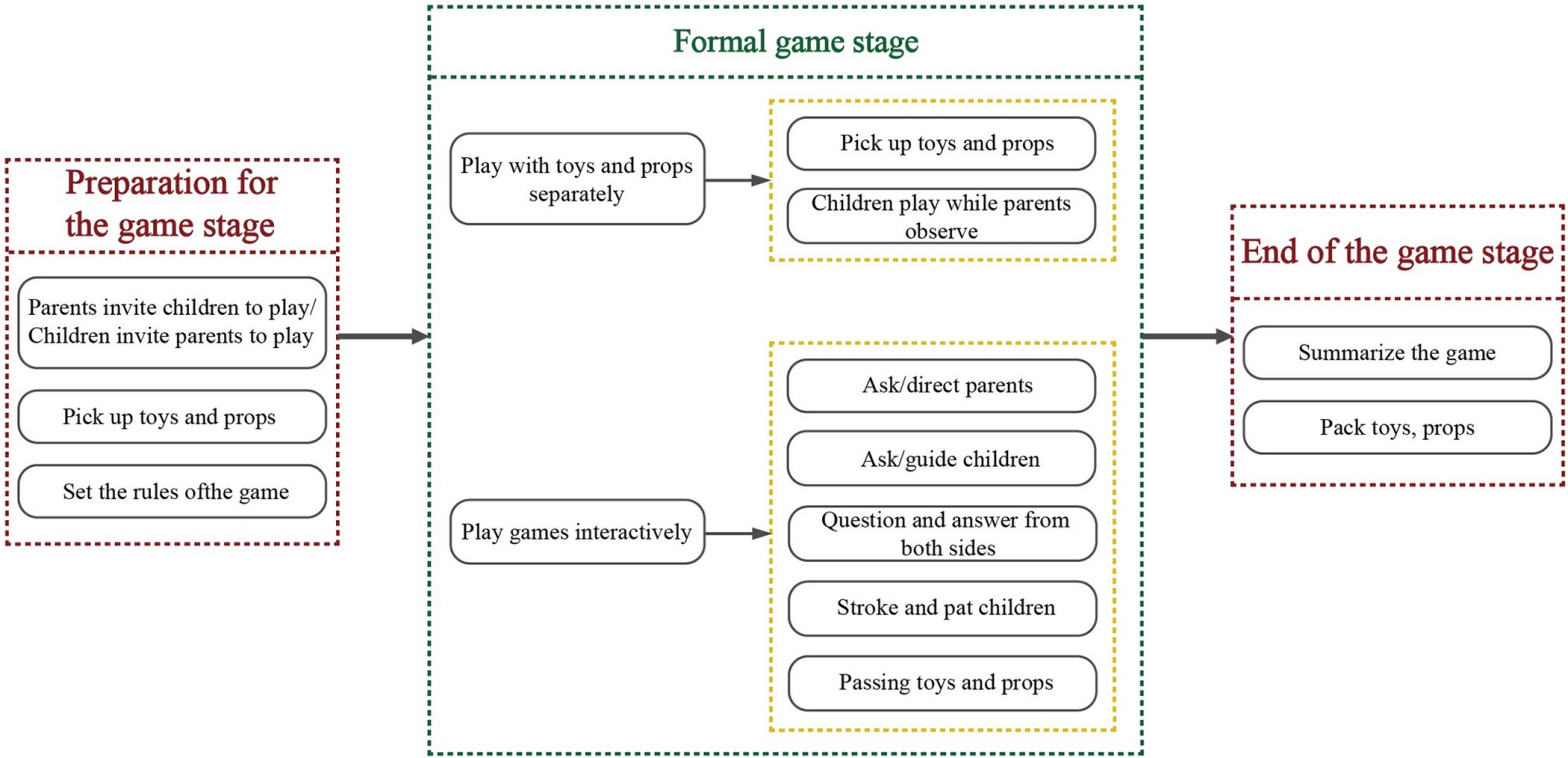
Flow chart of parent-child interactive games.

The observation method can record the parent-child play process and the behaviors generated by the users during the use process. Users’ needs for furniture are usually unconsciously manifested in body movements during use and interaction. In addition, regular summarization and motivational reasoning analysis of user behavior can help to discover the pain points of users’ needs and provide a basis for the subsequent extraction of user needs. By sorting out the game interaction process between parents and children, the entire process can be divided into three stages: the preparation stage, the formal game stage, and the game-ending stage. The whole process of parent-child interactive game is divided into three stages according to the time when the behavior occurs, and each stage of the game behavior occurs randomly, extinguishing rules and sequences (Fig 1).

The game preparation stage usually starts with the invitation of parents or children to play together. Before or after this, there is a possibility of picking up toys, and the general rules and content of the game will be planned. The initiator of the activity usually uses language as the medium for initiating invitations, and in most cases, children invite their parents to play with them. During the formal play phase, parents and children mainly exhibit two types of game behaviors. One is to play with toys and tools separately. In this state, there is no direct interaction behavior, usually manifested as non-contact and following the rules to jointly complete a game project or children’s game while parents observe from the side. The other type is direct interaction, which includes various direct interaction ways such as language and physical contact. In families undergoing household observation, one type of family engages in parent-child play by sitting on a mat and playing on the ground. Children’s sitting posture is unstable, with their legs and feet swaying freely and occasionally standing up and squatting. At the same time, boys tend to move better than girls, lying on the ground and rolling. The reason is that children are in a period of rapid development of both gross and fine movements, requiring much exercise to exercise themselves. Another type of family uses table and chair furniture for parent-child play, where the child sits in a chair, and the parent often adopts a sitting or squatting position on the floor in addition to a small chair to play with the child, possibly to make it easier to move around and interact with the child at any time. At the end of the game, children and parents often summarize the game behavior they just played. For example, they summarize the wins and losses in the game and encourage and praise children for completing paintings, game collages, etc. And finally, parents and children organize and tidy up game toys and props together.

After observing and analyzing the whole process and scenario of parent-child play interaction, it was found that the contact points between parents and children and the furniture revolve around fetching, playing, and storing, corresponding to the three stages of the play process.

The observation method helps to analyze user behavior and its motivation and reason out the corresponding user needs. However, when examining the user’s inner feelings through behavior is impossible, it is necessary to obtain valuable information in the interviews described by the user. The interview questions are set hierarchically according to the three phases of fetching, playing, and storing. In the fetch phase, questions were asked about the order and manner in which the game unfolded, the problems encountered during the game, and how the toys were picked up and placed. During play, ways of parent-child interaction, feelings about the use of the furniture, the positions taken, and their comfort were explored, and possible inconveniences during play were asked about. For the storage phase, the focus was on understanding children’s storage of toys, including the initiative to store children’s toys and the logic and rationale for categorizing toys. Combining the analysis of user behavior and the information obtained from the interviews, the user’s pain points were divided into three dimensions, as shown in Table 3. The primary dimension is the attributed category of the need, the secondary dimension is the refined attributed category after extracting the pain points, and the tertiary dimension is the enumeration of the joint pain points of different users in the interviews.

**Table 3:** Analysis of interview findings.

Primary dimension	Secondary dimension	Tertiary dimension
**Behavioral needs** **(access to toys)**	Storage space	Increased storage space to organize certain toys
	Sorting toys for storage	Difficulty finding the exact toy or prop to use in the locker
	Children’s Comfort in Accessing Toys	Children sometimes can’t reach them when accessing toys and sometimes kneel to clean them up.
**Behavioral Needs** **(play process)**	Satisfaction with manipulative space	Insufficient manipulative space for painting games, mind games, etc.
	Satisfying parent’s comfort	Parents’ legs and feet are not straight and their backs are curved.
	Satisfying children’s comfort	Ensure children’s comfort when using the equipment and do not harm their bodies.
	Meet children’s behavioral characteristics	Children do not like to sit properly on the chair to play, and like to crawl on the floor.
	Requirement for easy cleaning	Children may put paintbrushes on furniture, which is difficult to clean and erase.
	Blocking toys from falling	Fragmented toys and assembled parts often fall on the floor and cannot be found while playing.
	Meet the need for temporary storage	Some props in the course of play have no place to put them when they are not in use, and they are casually spread out on the side.
	Multi-scenario use	Simultaneously meets the three scenarios of children’s own games, games with peers, and games with parents.
	Demand for displaying results	No platform for displaying completed models and paintings
	Requirement for easy cleaning	Children may put paintbrushes on furniture, which is difficult to clean and erase.

### User behavioural requirements

We extracted the user’s behavioral needs based on the user’s pain points analyzed by the observation method and user interviews, as well as the posture preferences during parent-child games derived from the questionnaire, and the results are shown in Table 4.

**Table 4:** User behavior requirements description and keywords extraction.

Behavior content	Product requirements	
**Fetch**	Classify and store different toys for easy searching.	Classified storage
	There is a certain basis for classification and identification, making retrieving it convenient and fast.	Classification lables
	The lower storage space matches the height of children’s sitting and retrieval. The storage space on the last or second floor corresponds to the height of children bending down to retrieve items. Children do not need to pad their feet or lift their hands to reach for it, as it is not tiring to reach.	Suitable retrieval height for children’s human-machine size
	Not easy to tip over	Firm and stable
	Suitable for laying flat on paper.	Layable drawing paper
	The brush extends beyond the paper and can be erased when drawing on the table.	Easy to clean
	Control toy components to prevent them from falling out of the table boundary.	Prevent toys from falling
	Provide a plug-in base for Lego toys.	Docking base
**Play**	There is enough space to place intelligence cards, toys, etc.	Adequate operating space
	It can ensure the safety of children climbing, crawling, and drilling.	Safe to climb, crawl, and drill
	The hole space is sufficient for children to drill and crawl.	Suitable hole size
	Create a simulated scene and bring children into a situational game.	Scene simulation
	Being able to complete games on the ground is loved by both parents and children.	Play on the ground
	Make it comfortable for both children and parents to use.	Comfortable to use
	Play in the form of placing toys face-to-face in the middle.	Face to face play
	There is a certain basis for classification and identification, which can be classified when organizing.	Classified storage
**Store**	Children can easily reach the top area.	Suitable storage height for children’s human-machine size
	The posture of tidying up toys is comfortable.	
	A specific display function needs to display the results of children’s games.	Achievement display
	Store items that are not currently used but will be used later.	Temporary storage
	Children can easily reach the top area.	Suitable storage height for children’s human-machine size

### Quantify user behavior needs

According to the requirements of AHP and entropy weight evaluation method, experts in the furniture design industry and users of parent-child play furniture were invited to complete the questionnaire using 1–9 degree scale data and the Likert scale 7-level evaluation grade comment set. The AHP weights were then calculated using the 1 to 9 scale scores. The entropy weights were calculated using the statistical analysis results of the rubric set data. The results of the two data were combined to obtain a comprehensive ranking of essential indicators.

### AHP weighting and results

#### Step 1

**Fig 2:**
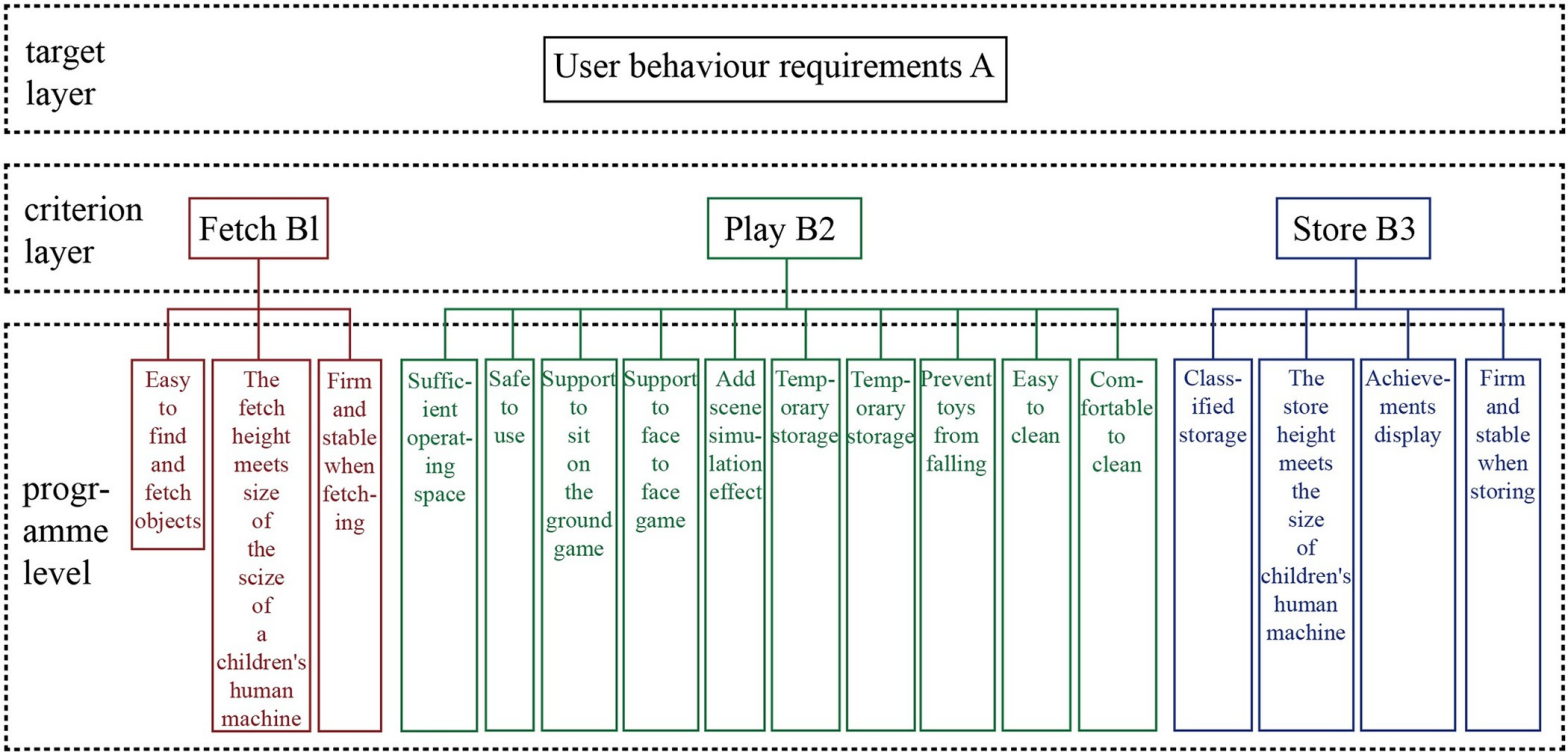
A hierarchical analysis model for user behavior requirements.

Establish a hierarchical structure. The number of user behaviour requirement items analyzed earlier is relatively large, so five furniture design students were selected to collate user behavioural demand keywords using the KJ method. Finally, 16 behaviour requirement layer indicators were obtained from three scenarios to construct an indicator system for user behaviour needs. The target layer is the user behaviour demand factor. The criterion layer is the three scenario factors of fetch, play, and store. The sub-criterion layer is the detailed requirements extracted from the three scenarios (Fig 2).

#### Step 2

Construct the judgment matrix. We invited 20 experts and designers in this research field to rate the quasi-criteria and sub-criteria layers according to the 1–9 scales method. The meanings of each scale in the 1–9 scales method are shown in Table 5. According to the AHP operation formula, the matrix, weight calculation, and consistency test were conducted for the criterion layer, ‘fetch’ sub-criterion layer, ‘game’ sub-criterion layer, and ‘storage’ sub-criterion layer, respectively. Tables 6–9 show the consistency test results, and the combined weights of the criterion layer and sub-criterion layer are shown in Table 10.

**Table 5:** Meaning of importance level of user demand factors.

Scale	Meaning
**1**	Ci element is as important as Cj element
**3**	Ci element is slightly more important than Cj element
**5**	Ci element is more important than Cj element
**7**	Ci element more important than Cj element
**9**	Ci is more important than Cj.
**2,4,6,8**	Median of two adjacent judgments
**1, 1/2,. . ., 1/9**	The ratio of the importance of the element Ci over the element Cj is the reciprocal of the above

**Table 6:** Criteria layer matrix, weights, and consistency test results.

	B1	B2	B3	Weight	Consistency indicator (CI)	Inspection coefficient (CR)
**B1**	1	1/3	1	0.2000	0.0000	0.0000<0.10 Pass
**B2**	3	1	3	0.6000		
**B3**	1	1/3	1	0.2000		

**Table 7:** Matrix, weight, and consistency test results of the ‘fetch’ sub-criterion layer.

Fetch	C1	C2	C3	Weight	Consistency indicator (CI)	Inspection coefficient (CR)
**C1**	1	1/2	1/5	0.1220	0.0036	0.0007<0.10 Pass
**C2**	2	1	1/3	0.2297		
**C3**	5	3	1	0.6483		

**Table 8:** Matrix, weight, and consistency test results of the ‘play’ subcriteria layer.

Play	C4	C5	C6	C7	C8	C9	C10	C11	C12	Weight	Consistency indicator (CI)	Inspection coefficient (CR)
**C4**	1	1/5	1/3	1/2	5	3	5	5	1/5	0.0831	0.0693	0.0478< 0.10 Pass
**C5**	5	1	3	3	5	4	7	7	3	0.2943		
**C6**	3	1/3	1	3	5	5	7	7	1/3	0.1603		
**C7**	2	1/3	1/3	1	3	2	4	4	1/5	0.0849		
**C8**	1/5	1/5	1/5	1/3	1	1/3	1/2	1/2	1/7	0.0243		
**C9**	1/3	1/4	1/5	1/2	3	1	2	2	1/6	0.0453		
**C10**	1/5	1/7	1/7	1/4	2	1/2	1	1	1/7	0.0269		
**C11**	1/5	1/7	1/7	1/4	2	1/2	1	1	1/7	0.0269		
**C12**	5	1/3	3	5	7	6	7	7	1	0.2540		

**Table 9:** Matrix, weight, and consistency test results of the ‘store’ subcriteria layer.

Store	C13	C14	C15	C16	Weight	Consistency indicator (CI)	Inspection coefficient (CR)
**C13**	1	1/2	3	1/5	0.1368	0.0274	0.0308<0.10 Pass
**C14**	2	1	3	1/2	0.2159		
**C15**	1/3	1/3	1	1/7	0.0653		
**C16**	5	2	7	1	0.5820		

**Table 10:** The weight values of each matrix in the AHP.

Primary indicators	Weight	Secondary indicators	Weight	Comprehensive weight	Ranking
**Fetch**	0.2000	Easy to find and fetch objects	0.1220	0.0244	12
		The fetch height meets the size of a children’s human machine	0.2297	0.0459	8
		Firm and stable when fetching	0.6483	0.1297	3
**Play**	0.6000	Sufficient operating space	0.0831	0.0499	7
		Safe to use	0.2943	0.1766	1
		Support to sit on the ground game	0.1603	0.0962	5
		Support to face to face game	0.0849	0.0509	6
		Add scene simulation effect	0.0243	0.0146	15
		Temporary storage	0.0453	0.0272	11
		Prevent toys from falling	0.0269	0.0162	13/14
		Easy to clean	0.0269	0.0162	13/14
		Comfortable for both parties to use	0.2540	0.1524	2
**Store**	0.2000	Classified storage	0.1368	0.0274	10
		The store height meets the size of a children’s human machine	0.2159	0.0432	9
		Achievements display	0.0653	0.0131	16
		Firm and stable when storing	0.5820	0.1164	4

### Entropy weighting and results

#### Step 1

Construct the original judgment matrix. A five-level Likert scale was used to design the Parent-Child Interactive Game-based Furniture User Behavioral Needs Questionnaire, and the 16 need factors at the sub-criteria level were taken as the core questions of the questionnaire. The corresponding questions were designed for each influencing factor separately. The questionnaire consists of two parts: the basic information of the subjects and the specific questions involved in the influencing factors. This study evaluated the questionnaire before a large amount of questionnaire data was formally collected. Five furniture design industry experts and five children’s play furniture users were invited to pre-test the questionnaire, and the questionnaire was modified to address common problems such as ambiguity of words, presentation errors, and possible ambiguity of content. Based on their suggestions, the questionnaire was redesigned using a seven-point Likert scale to obtain more detailed quantitative data on users’ psychological responses, and the questionnaire was finally obtained. Specific questions in the questionnaire ask about the degree of user need for a demand factor, V = {Very little need, no need, relatively little need, indifferent, relatively need, need, very much need}, corresponding to a score of 1–7 points, the user needs to choose a certain degree of scoring. The questionnaires were distributed to parents of children between the ages of 3 and 6 and collected online and face-to-face. Eighty questionnaires were distributed offline and were collected after being filled in face-to-face. Fifty questionnaires were distributed online, mainly to parents of 3-6-year-olds who were unable to fill in the questionnaire face-to-face, and the rules for completing the questionnaires were explained in detail before completing the questionnaires. In addition, before determining the weights, it is necessary to decide on the direction of the influence of the indicators on the target scores and to pay attention to the influence of extreme values so as to pre-process or exclude the indicators that are non-linear. After excluding nine invalid questionnaires, 121 valid questionnaires were counted with a validity rate of 93% and a Cronbach ɑ’s value of 0.837 (greater than 0.8), which implies that the obtained data have excellent reliability. The results of the statistical analysis of the comment set data were used to construct the original matrix for the indicator layer. The arithmetic mean of the user ratings of the indicators included in the criteria layer is used to build the initial matrix of the criteria layer.

#### Step 2

Calculate the objective weights. Due to a large amount of scoring data, we used the SPSSAU software to calculate the entropy weights according to the calculation steps of the entropy weight method of Eq (5) to Eq (8). The original matrix of the indicator layer and the original matrix of the criterion layer is imported into SPSSAU. The software obtains the weight coefficients of the criterion layer and the factors of the indicator layer after calculating the criterion according to the entropy weight method. The weight of the indicator layer is multiplied by the weight of the corresponding criterion layer to finally obtain the comprehensive, objective weights of the indicator factor. The specific results are shown in Table 11.

**Table 11:** The weight values of each matrix in the entropy weight method.

Primary indicators	Entropy weight coefficient	Secondary indicators	Entropy weight coefficient	Comprehensive weight	Ranking
**Fetch**	0.4805	Easy to find and fetch objects	0.0973	0.0467	6
		The fetch height meets the size of a children’s human machine	0.1170	0.0562	3
		Firm and stable when fetching	0.7857	0.3775	1
**Play**	0.2338	Sufficient operating space	0.0673	0.0157	11
		Safe to use	0.2784	0.0651	4
		Support to sit on the ground game	0.1500	0.0351	10
		Support to face to face game	0.0748	0.0175	9
		Add scene simulation effect	0.0645	0.0151	15
		Temporary storage	0.0681	0.0159	14
		Prevent toys from falling	0.0730	0.0171	12
		Easy to clean	0.0696	0.0163	13
		Comfortable for both parties to use	0.1542	0.0361	8
**Store**	0.2857	Classified storage	0.1752	0.0500	7
		The store height meets the size of a children’s human machine	0.2219	0.0634	5
		Achievements display	0.2083	0.0595	16
		Firm and stable when storing	0.3946	0.1127	2

### Comprehensive weights

**Fig 3:**
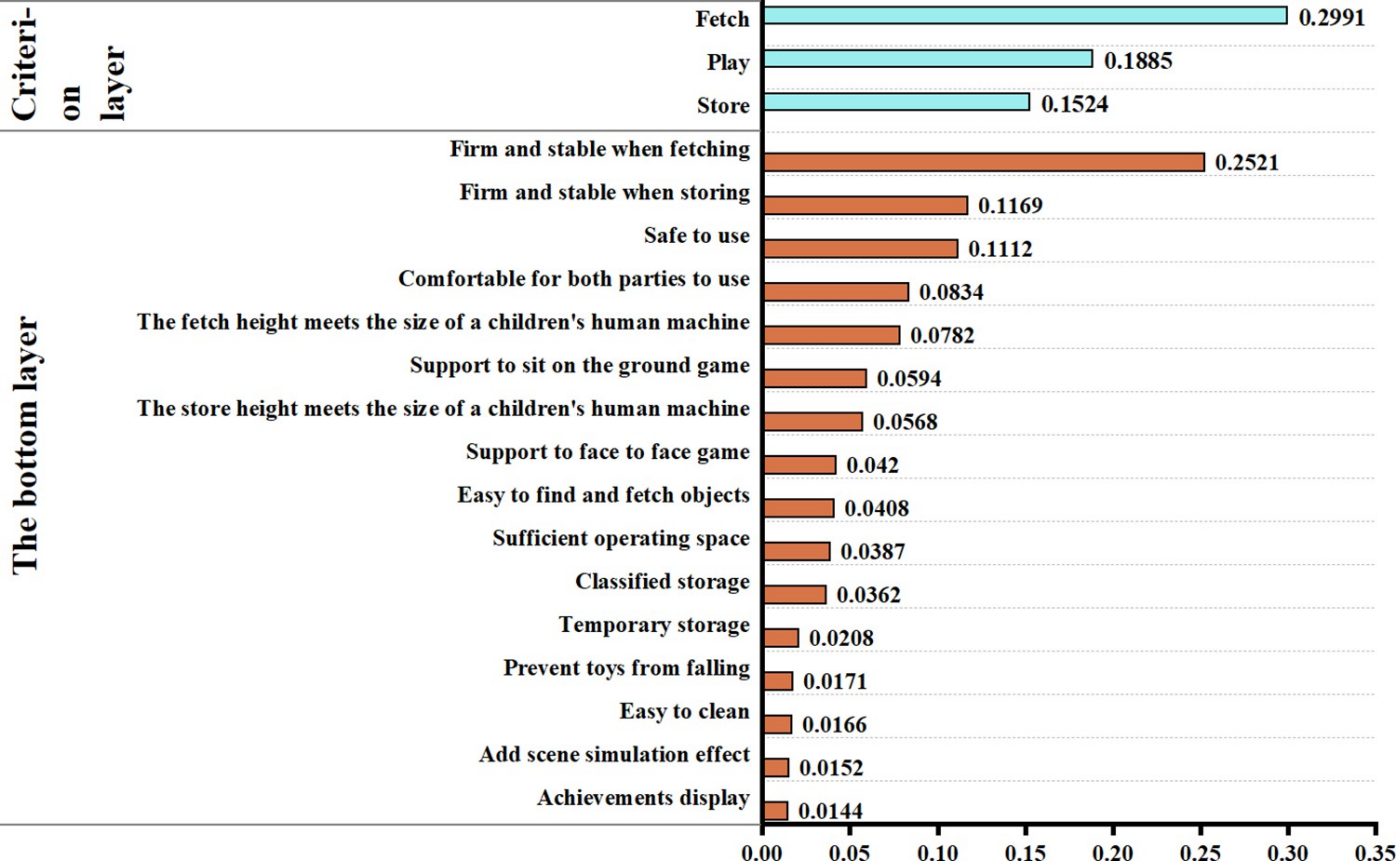
Comprehensive weights ranking of user behaviour requirements.

Comprehensive weights are calculated and ranked based on the subjective and objective weights obtained from Eq (9) (Fig 3). In Eq (9), ɑ is the proportion coefficient, indicating that the proportion of subjective and objective weights needs to be balanced when assigning weights. In this study, to fully reflect the objective decision-making role of the user in design evaluation, design industry experts recommend 0.4.

## Discussion

In this study, we collected and analyzed behavioral preferences during parent-child game interactions, as well as interactive behaviors between parents and children and between users and furniture, through questionnaires, household observations, and interviews. On this basis, we refined the behavioral demand for furniture in parent-child game interaction. We used the AHP-entropy weighting method to rank the comprehensive importance of the extracted user behavioral requirements. To better acquire user needs, we conducted market research before the user study, categorized children’s game furniture in the market according to the main form of use, and analyzed the game functions provided by each type of furniture according to the classification criteria of children’s games in this study, and found that the types of games that the products can satisfy have a strong correlation with the main form of use. This section discusses the results of the user research as well as the results of the user behavioral demand weights obtained through the AHP-entropy weighting method.

### Analysis of parent-child interaction behaviour

User behavioral requirements extraction takes, to some extent, the information obtained in the questionnaire. We designed relevant questions in the questionnaire to understand the game location, game interaction duration, game position status, parent-child game posture, types of toys that parent-child often play, and the correlation between parent-child interaction game types and age. The relevant questions in the questionnaire were analyzed after using frequency statistics, multiple response frequency analysis, and cross-tabulation chi-square test.

Regarding the duration of parent-child interactive games, it can be found that 2.6% of children have a duration of 0–15 minutes, and 19.2% have a duration of more than 45 minutes. The frequency of interaction duration between 15–30 minutes and 30–45 minutes is relatively high, reaching 39.1%, indicating that most parent-child interaction games in families grow up within the range of 15–45 minutes, significantly longer than children playing alone. At the same time, through a cross-table chi-square test, the relationship between game duration and children’s age was found to be P>0.05, indicating no significant correlation between parent-child game duration and children’s age. Parent-child interaction time of over 15 minutes is the norm in most families, meaning users have a certain length of time in contact with furniture. During this period, parents accompany children and play together. Therefore, the comfort of parents and children’s posture is an essential factor in the design.

Regarding the selection of parent-child game positions, the most frequent one is in face-to-face game positions, accounting for 46.8% of the population, followed by game positions on the same side, accounting for 40.4%, with fewer options for right angles and placing children on their bodies. Regarding the survey of game posture preference, parents were surveyed using text questions in the questionnaire, and children were matched with pictures. From the questionnaire results, it can be seen that parents’ favourite interactive posture is sitting on the ground, accounting for 56.4%, followed by sitting on the sofa (28.2%), sitting in a chair (14.1%), and standing (1.3%). The favourite parent-child interaction posture for children is also sitting on the ground, accounting for 40.4%, followed by sitting in a chair (26.3%), standing (19.9%), and sitting on the sofa (13.5%). The research results show that parents and children prefer to sit on the ground and play with each other, and parents are even more inclined to play parent-child games on the ground than children. However, the proportion of children who enjoy playing in chairs and standing games is higher than those who enjoy sitting on sofas, while parents are the opposite. The reason is closely related to the height and size of the seat. A seat height that is too high can widen the gap with a child’s height, while a seat height that is too low does not meet the ergonomic dimensions of an adult. Fixed seats cannot be moved freely, making it more convenient for parents to move while sitting on the ground.

From the selection of the seven major types of games, it can be seen that there are differences in the selection of parent-child games among the three age groups: children aged 3–4 tend to choose painting and handicraft games (72.1%), sports games (72.1%), and music/performance games (67.6%), while they have relatively fewer choices for games that rely on tools or toys; Children aged 4–5 often choose painting and handicraft games (72.1%), intelligence games (62.8%), and structural games (62.8%) as their parent-child games; At the age of 5–6, people tend to choose more famous painting and handicraft games (82.2%), structural games (82.2%), and also choose more intelligence games (66.7%). Relatively consistent is that painting and handicraft games have the highest choices in all three age groups. As age increases, intelligence games and structural games are more commonly chosen, while the choice of sports games gradually decreases. Considering the frequency of toy use and parent-child interactive game selection results, corresponding to the type of children’s game furniture, the table and chair furniture can meet the needs of most users.

From the combined weights, the weights were mainly in favor of meeting safety and comfort needs. In addition, the need for play posture status and size setting to meet the posture of fetching and storing objects and providing sufficient play space is also essential. On the other hand, preventing toys from falling, being easy to clean, and having scene simulation effects and display results can be considered additional needs and are optional. The material of children’s play furniture has a significant impact on comfort and ease of cleaning. More soft elements need to be incorporated into children’s play furniture, and upholstered seating surfaces should be designed to be easily replaceable wherever possible. Users’ concern about the safety of play furniture lies mainly in the process of children holding and placing toys. The stability of children’s storage furniture structures needs to be strengthened, and lightweight materials and rounded corners should be used as much as possible. Parents want their children to develop good toy storage habits, so the storage design should align with children’s human-machine dimensions to facilitate children’s use. During play, except for drawing and other games that must be played in a seated position with a tabletop, parents and children like to sit directly on the floor to facilitate interaction and a comfortable experience. When designing the relevant furniture, consider replacing the matching seats with cozy, soft, easy-to-move cushions. Finally, at the end of the game, children may want to show their parents what they have accomplished. Therefore, the design part of the play table should be a small chalkboard to facilitate communication and sharing between children and their parents.

Overall, the Analytic Hierarchy Process (AHP) and the Entropy Weight Method (EWM) have high weights for factors related to comfort and safety. In the AHP, scores are given higher relative importance, while in the EWM, users always choose highly compatible options regarding safety and comfort-related issues. The influence factors with lower weights obtained lower relative significance in the AHP, while in the EWM, it is due to the significant differences in user evaluations of the significance of these influence factors.

The AHP-entropy weight method can retain the objective evaluation of the user group and the subjective judgment of industry experts, reflecting the scientific nature of combining subjective and objective weighting. The evaluation model provides a theoretical basis and quantitative indicators for improving the functional design of parent-child interactive play furniture and plays an essential role in enhancing user satisfaction with the product.

## Conclusion

This study takes meeting user behavioral needs as the starting point. Through market research, it analyzes the status quo of parent-child interactive game furniture-related products to provide a reference basis for user demand mining. Questionnaire research method, observation method, interview method, and other methods are used to refine the user behavioral needs of parent-child interactive game furniture. The hierarchical analysis method combined with the entropy weight method is used to analyze the subjective and objective comprehensive weights of user behavioral needs. The proposed evaluation model can accurately determine users’ behavioral needs and importance when using parent-child interactive game furniture, providing practice, theoretical basis, and data support for the design research of parent-child interactive game furniture products. The subjective-objective combination of the fuzzy comprehensive evaluation model proposed in this study incorporates both the views of industry experts and users of parent-child interactive game-based furniture. In contrast, children’s game furniture design often focuses more on meeting children’s needs and ignores parents’ expectations. Our model bridges this gap by balancing the weight of children’s and parent’s needs, ensuring that designs are more inclined to meet the needs of parents and children for joint use. This model also applies to other furniture design studies to avoid designers’ over-reliance on past experiences, understand users’ goals and actual needs, and synthesize these two perspectives to rank the weights of design elements. When researching and analyzing the behavioral needs of parent-child users, we discovered a critical point the market has overlooked: parents and children tend to adopt a sitting position when playing together. Parent-child interactive game furniture in the market is mainly equipped with short stools, so in future design, we should consider adopting the form of sitting on the floor, which is more in line with users’ needs. In addition, users have higher requirements for the rationality and convenience of game-based furniture’s operating space and storage space. In design practice, more attention should be paid to the rational configuration and division of the use and storage space.

The entropy weight method relies entirely on indicator values when calculating objective weights. For the same indicator, the weight naturally changes as long as the indicator value changes. The weight becomes more stable as more questionnaires are collected and better reflect user satisfaction. We can consider increasing the sample number and geographical breadth in future research. For more detailed additional elements such as storage guidance and personalization, further study and exploration can be conducted to meet better the basic functional, interactive, and emotional needs of parents and children.

## Supporting information

S1 File The raw data which is required for entropy weighting calculations. (XLSX)
